# Possible Diagnostic and Therapeutic Applications of Bioprinting for Bone Regeneration in Maxillofacial Surgery

**DOI:** 10.3390/diagnostics15232978

**Published:** 2025-11-24

**Authors:** Lorenzo Marini, Alessandro Tel, Marco Zeppieri, Luca Michelutti, Massimo Robiony, Caterina Gagliano, Fabiana D’Esposito, Matteo Capobianco, Tamara Ius, Marieme Khouyyi

**Affiliations:** 1 Clinic of Maxillofacial Surgery, Head-Neck and NeuroScience Department, University Hospital of Udine, Piazzale S. Maria della Misericordia 15, 33100 Udine, Italymicheluttiluca.uniud@gmail.com (L.M.);; 2 Department of Ophthalmology, University Hospital of Udine, 33100 Udine, Italy; 3 Department of Medicine, Surgery and Health Sciences, University of Trieste, 34127 Trieste, Italy; 4 Department of Medicine and Surgery, University of Enna “Kore”, Piazza dell’ Università, 94100 Enna, Italy; 5 Eye Center “G.B. Morgagni-DSV”, 95125 Catania, Italy; 6 Imperial College Ophthalmic Research Group [ICORG] Unit, Imperial College, London NW1 5QH, UK; 7 Eye Clinic, Catania University, Policlinico G. Rodolico, Via Santa Sofia, 95121 Catania, Italy; 8 Faculty of Medicine, University of Catania, 95123 Catania, Italy; 9 Academic Neurosurgery, Department of Neurosciences, University of Padova, 35121 Padova, Italy; 10 Department of Biomedical and Dental Sciences and Morphofunctional Imaging, University of Messina, 98121 Messina, Italy

**Keywords:** bioprinting, craniomaxillofacial surgery, bone tissue, regenerative medicine, bone regeneration

## Abstract

**Background**: The integration of 3D bioprinting, biomaterials science, and cellular biology presents a viable strategy for maxillofacial bone regeneration, overcoming the constraints of traditional graft techniques. This review offers a thorough examination of the present condition, obstacles, uses, and future outlook of 3D bioprinting technology in maxillofacial bone regeneration. An essential understanding has been attained by analyzing the technological constraints, specifically in vascularization and neuro-integration, and by delineating the vital translational pathway from benchtop models to clinical application. We have examined several bioprinting techniques—namely extrusion, inkjet, and laser-assisted methods—and the requisite bioinks, emphasizing their physicochemical and biological features vital for osteogenesis. Significant clinical applications, including the treatment of trauma-induced abnormalities and the reconstruction of oncology-related resections, have been emphasized. This review highlights the urgent necessity for established regulatory frameworks and refined printing settings to guarantee effective, functional, and durable bone substitutes, providing a distinct pathway for future research and clinical implementation in this specialized surgical domain. **Aim**: The purpose of this review was to present a general overview of the current clinical and diagnostic applications of bioprinting in bone tissue engineering for the reconstruction of bone defects. **Methods**: A search of major scientific databases, including PubMed, Science Direct, Embase, and Cochrane, was conducted. Articles published within the last 10 years that analyze the possible applications of bioprinting in bone tissue fabrication were included. **Results**: Several bioinks, based on hydrogels and stem cells, can enable the fabrication of such tissues using this technology. This review reports on the processes adopted for the bioprinting of bone tissue, the bioinks used, and cell cultivation methods. **Conclusions**: Bioprinting represents a promising solution for bone regeneration with potential applications that could revolutionize current surgical practices, despite the many challenges that future research will face.

## 1. Introduction

### 1.1. Background of Bioprinting

Three-dimensional bioprinting is an innovative technology that leverages three-dimensional printing techniques to create biological structures, such as tissues and organs, using special inks, bioinks, and living cells. This printing technique enables the creation of three-dimensional biological constructs useful for regenerative medicine studies, personalized surgery, and drug research. Unlike traditional 3D printers, this technology uses living cells, biomaterials, and biomolecules, including growth factors, to create biological tissues and structures, with the aim of reporting the functionality and characteristics of native tissues [1,2,3].

Bioprinting, due to its capabilities, offers potential applications, from regenerative medicine to pharmacological studies. One of the main applications finds room in regenerative medicine, which deals with regenerating or replacing tissues damaged by disease processes or aging. The possibility of making biological constructs that mimic the composition of tissues such as cartilage, skin, bone, vessels, and muscle would provide a future opportunity to make potentially implantable tissues or organs, avoiding the need for transplants, the complications associated with them, including the risks of rejection, and alleviating donor shortages [4,5]. In addition, this technology enables the creation of disease models for drug trials, particularly for developing therapeutic strategies against oncological diseases. In fact, these bioprinted constructs can be used to test new drugs, accelerating the development of therapies and reducing the need for animal species trials [6].

**Figure 1:**
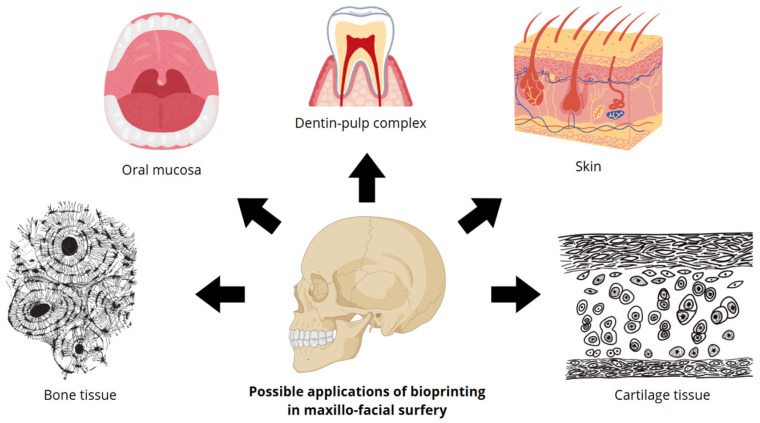
Schematic representation of possible applications of bioprinting in maxillofacial surgery. The main types of tissues studied in this medical field are represented.: bone tissue, oral mucosa, dentin–pulp complex, skin, and cartilage tissue.

With regard to the field of Maxillofacial surgery, bioprinting proves to be a potential future tool for the benefit of surgeons, particularly offering innovative solutions for the treatment of bone defects, traumatic injuries, congenital malformations, and lesions of mucosal skin and cartilaginous tissue (Figure 1) [7]. Figure 1 shows the potential applications of bioprinting in maxillofacial surgery. The primary tissue types examined in this medical discipline include bone tissue, mouth mucosa, dentin–pulp complex, skin, and cartilage tissue.

This innovative technology could be useful in the future for the reconstruction of complex anatomical areas subject to surgical demolition, particularly in patients who have tumor lesions to be removed. Currently, the gold standard for reconstruction of the anatomical regions subject to lesion removal, including tumor lesions, is surgical flaps, such as the temporalis muscle, fibula, or scapula flap [8]. The hypothetical and future possibility of reconstructing these anatomical areas by implanting bioprinted constructs would revolutionize current reconstructive surgery. Bioprinting represents a potentially useful tool for soft tissue regeneration, reconstruction of bone defects, fabrication of skin and cartilage tissues, and the study and testing of new therapeutic strategies, particularly pharmacological ones [9,10,11,12].

The fabrication of a bioprinted construct is a complex, multi-step process that extends from the initial design phase to post-printing maturation. The preparatory stage involves defining the objectives, function, and desired characteristics of the construct, followed by the creation of a digital 3D model using computer-aided design (CAD) software. Equally important is the selection of appropriate bioinks, biomaterials, and cell types, chosen according to the specific tissue to be replicated. Prior to printing, the bioprinter must be calibrated and loaded with the selected materials, and the overall architecture of the construct—both anatomical and histological—must be carefully analyzed.

During the printing phase, successive layers of bioink and living cells are deposited according to the pre-designed 3D model. Various bioprinting techniques can be employed, each offering distinct advantages and limitations depending on the tissue type. Once printing is complete, post-bioprinting procedures are performed to promote tissue maturation. The construct is typically placed in a bioreactor or controlled cell-culture environment to support cell differentiation, proliferation, and the development of functional tissue properties [13,14,15].

The purpose of this narrative review is to analyze the possible fabrication of bone tissue constructs through bioprinting and potential applications in the field of maxillofacial surgery. The most widely used biomaterials for bone tissue fabrication will be reviewed, focusing on their chemical-physical and mechanical properties, the most appropriate printing techniques, and the living cells adopted. Finally, the main limitations and future challenges for the development of bioprinted bone constructs will be addressed, particularly the creation of vascularized structures, a crucial element in ensuring tissue survival and function.

### 1.2. Methodology and Search Criteria

A comprehensive literature search was performed across prominent databases such as PubMed, Scopus, and Web of Science. The search method utilized a blend of Medical Subject Headings (MeSH) and free-text terms like “3D bioprinting,” “maxillofacial bone regeneration,” “tissue engineering,” “bioinks,” and “osteogenesis.” The search was limited to articles published from 2015 onward to encompass the latest advancements in the discipline. The inclusion criteria encompassed original research publications, reviews, and clinical trials that particularly addressed the application of 3D bioprinting techniques for bone deformities, bioink production, and cell viability studies pertinent to the maxillofacial region. The exclusion criteria encompassed papers not authored in English, those concentrating exclusively on conventional 3D printing (lacking cells/bioinks), and studies limited to non-skeletal tissues. Two independent reviewers assessed the titles and abstracts, followed by a full-text examination of selected publications to ascertain the relevance and quality of the included literature.

## 2. Bone Defects and Regeneration

### 2.1. Bone Defects in Maxillofacial Surgery

In maxillofacial surgery, it is common to encounter bone defects of various kinds. Bone defects are mainly due to trauma or surgery for tumors, infections, or developmental abnormalities. One of the anatomical areas most affected by bone defects is the mandible. The possible causes of mandibular bone defects are congenital diseases, tumors, trauma, inflammatory diseases, and bisphosphonate osteonecrosis (BRONJ), of which the most common are congenital malformations and acquired complications [16,17,18,19,20,21].

Autologous bone grafts, such as iliac or costochondral grafts, or allografts, bone marrow aspirate concentrate, or demineralized matrices are often used to treat such defects. In addition to these, it is possible to reconstruct the defect through the use of implantable plates and prostheses, such as custom prostheses made of porous titanium, a material that has good biocompatibility and implantability [22,23,24]. The gold standard is autologous bone transplantation, as it has low immunogenicity, low risk of rejection, and rapid osteogenesis and bone regeneration. On the other hand, allogeneic transplantation has a high risk of rejection and infection. However, the main advantage is the wide availability and absence of the donor site, unlike autologous transplantation. Finally, metallic prostheses, particularly those made of porous titanium, are widely used to reconstruct and repair bone defects, considering their mechanical strength, bone integration, durability, and the possibility of synthesis means and customized prostheses using virtual surgical planning software and 3D printing technology [8,25,26]. Table 1 lists the advantages and limitations of the various types of therapeutic options in the treatment of bone defects.

### 2.2. Biological Principle of Bone Regeneration

Bone regeneration is a fundamental physiological process that enables the recovery of structural and functional integrity of bone tissue following trauma or metabolic disorders. It involves a highly coordinated sequence of cellular and molecular events regulated by cytokines, growth factors, and adhesion molecules [27,28]. Understanding these mechanisms is essential for the development of regenerative strategies and tissue engineering approaches aimed at restoring bone continuity and function.

The regenerative cascade proceeds through four main stages: inflammatory, fibrocartilaginous, ossification, and remodeling. The inflammatory phase begins immediately after injury, when hematoma formation triggers an immune response involving neutrophils and macrophages. These cells release cytokines, pro-inflammatory mediators, and growth factors that initiate the healing process. During the subsequent fibrocartilaginous callus formation, fibroblasts and mesenchymal stem cells (MSCs) migrate to the damaged site and differentiate into chondrocytes, producing a cartilaginous matrix that acts as a temporary scaffold. This matrix is progressively replaced by mineralized tissue through the process of endochondral ossification, during which osteoblasts derived from MSCs deposit bone matrix and promote the formation of new bone. Finally, in the remodeling phase, osteoclasts resorb immature bone while osteoblasts form mature lamellar bone, restoring the original microarchitecture and mechanical properties [27,29].

The main cellular players in this process are osteoblasts, which synthesize and mineralize the bone matrix, osteoclasts (responsible for bone resorption), and MSCs, which provide the osteogenic and chondrogenic precursors essential for regeneration. Endothelial cells also play a crucial role, as the formation of a new vascular network ensures oxygen and nutrient supply and removal of catabolic products, supporting tissue survival and integration [30].

At the molecular level, bone regeneration is governed by a complex interplay of signaling pathways and growth factors [31]. Among these, Bone Morphogenetic Proteins (BMPs) induce MSC differentiation into osteoblasts and are widely investigated for therapeutic use. Parathyroid Hormone (PTH) regulates bone metabolism and stimulates osteoblastic activity, while the Wnt/β-catenin pathway promotes osteoblast proliferation and differentiation. In parallel, Notch signaling contributes to the maintenance and renewal of MSC populations and regulates their osteogenic differentiation [32].

### 2.3. Fundamental Properties of Bioprinted Bone Tissue Constructs

Bioprinted bone tissue constructs are much more complex than other constructs, such as those for skin, cartilage tissue, or mucosa. In fact, the fabrication of such tissue still has many limitations and challenges to overcome, such as making a performant vascular network. For the realization of bone tissue, it is essential to meet certain characteristics that underlie it. For several years, the “diamond concept” has been recognized as a diagram that outlines the essential elements for achieving good bone regeneration. This conceptual framework includes: sufficient stabilization and mechanical strength; osteogenic cells; scaffolds and osteoconductive structures; growth factors; and adequate vascularization of the newly formed tissue [32,33].

Bioprinted tissues are designed to mimic the biological, mechanical, chemical-physical, and structural properties of native bone, with the purpose of repairing or replacing tissue damaged as a result of bone defects or metabolic-degenerative diseases.

**Mechanical properties**. Bone tissue is characterized by significant resistance to mechanical stress. Bioprinted constructs to replicate these properties must exhibit stiffness and compressive strength, elasticity, and controlled porosity. The latter is a critical aspect for the fabrication of spongy bone; in fact, porosity enhances the ability to support bone remodeling, but also allows vascularization of the tissue, a crucial aspect for the process of bone regeneration [34,35].

**Biological properties**. Bioprinted bone constructs must have properties to promote integration with native bone tissue and stimulate bone regeneration. These properties are: biocompatibility, induction of cell differentiation, vascularization, adhesion, and cell proliferation. These bioprinted tissues must be made from biocompatible biomaterials, have no immunogenicity, and be capable of promoting integration with adjacent tissues. They must also contain molecular signals that induce cell differentiation, such as BMPs, and include stem cells, such as MSCs, to stimulate the formation of bone neo-tissue. A further aspect is the establishment of a functional vascular network, which is crucial for the survival and success of these constructs. Through vascularization, it is possible to bring in nutrition and oxygen, remove catabolic wastes, and allow access of immune system cells for a defense function. Finally, the biomaterials used must promote stem cell adhesion by supporting cell proliferation [36,37,38,39].

**Structural properties**. Additional properties that these bioprinted constructs must have to properly ensure their mechanical and biological functions are geometry, fiber orientation, and camouflage of bone microarchitecture. In fact, the geometry of bioprinted tissues is crucial because they must be tailored to each case, adapting the shape and size with respect to the specific defect. Fiber orientation also plays an important role, as it can affect the direction of cell growth and the mechanical strength of the tissue. In addition, it is critical to replicate the microarchitecture of native bone tissue to optimize mechanical stress distribution and vascularization [40,41].

**Chemical properties**. The biomaterials that are used to make these bioprinted bone constructs must have a chemical composition more similar to that of the native tissue. In fact, the most commonly used materials are hydroxyapatite, tricalcium phosphate, and synthetic or natural polymers. The choice of the type of biomaterial depends greatly on the characteristics required for the construct. Nowadays, there is a tendency to use bioinks consisting of several complementary biomaterials in order to satisfy a larger number of properties. Finally, these constructs must be biodegradable so that they can be slowly replaced by new bone tissue formed by the natural process of bone regeneration [42,43,44].

## 3. Bioinks

Bioinks are materials used to create the bioprinted construct. They are biocompatible materials, often hydrogel-based, that contain living cells, biomaterials, biomolecules, and growth factors. These particular inks must possess special characteristics in order to ensure cell survival, differentiation, and functional tissue formation, taking into consideration the specific properties of the tissue to be printed [45,46].

### 3.1. Biomaterials and Fundamental Properties

The biomaterials that make up bioinks can be natural (such as collagen, alginate, hyaluronic acid) or synthetic (such as PEG or PLA). Often, in order to optimize mechanical, physicochemical, biological, and functional properties, bioinks are created consisting of multiple types of biomaterials that complement each other [47].

The key properties of bioinks are: biocompatibility, printability, mechanical properties, biodegradability, and biological functionality. In fact, they must support cell proliferation and survival without triggering adverse immune responses (biocompatibility), they must have proper viscosity to ensure extrusion and maintain the desired shape of the construct after deposition (printability), they must resist mechanical stresses (mechanical), they must degrade in a controlled manner (biodegradability), and they must have molecular signals within them in order to ensure cell differentiation and tissue formation (biological functionality) [4,38,48,49,50].

### 3.2. Biomaterials Suitable for Bone Bioprinted Constructs

Taking into consideration the biological principles of natural bone regeneration and the properties to be met by bioinks for bone tissue the main biomaterials used for making bioprinted bone constructs are hydroxyapatite (HA), a ceramic material that mimics the mineral component of bone by presenting good biocompatibility and osseointegration ability; beta-tricalcium phosphate (β-TCP), a biodegradable ceramic material that, presenting good resorb and osteoconductive properties, is often combined with hydroxyapatite; polyacid-lactic acid (PLA) a synthetic biomaterial with rapid resorption; poly-capro-lactone (PCL), a synthetic biomaterial with good resistance to long-term mechanical stress; and gelatin, a natural biopolymer derived from collagen that is characterized by good biocompatibility and cell-supporting capacity and is able to promote cell adhesion and metalloproteinases (MPPs), alginate, a natural biomaterial often used to form hydrogels and suitable for making porous structures, collagen, a natural biomaterial useful for increasing osteogenic differentiation and cell adhesion [50,51,52,53,54,55].

Often, these biomaterials are combined to achieve improved mechanical and biological properties, for example, by combining ceramic materials (HA, β-TCP) and natural or synthetic polymers (PLA, PCL). A widely used compound biomaterial is methacryloyl-modified gelatin (GelMA), which is characterized by biodegradability, nonimmunogenicity, biocompatibility, and good cell adhesion. Often this biomaterial is used as an expendable material for making canalicular structures in order to promote nutrient and oxygen diffusion, facilitating cell differentiation and proliferation [56,57,58].

## 4. Living Cells

Living cells are another key element of bioinks. They can be present directly in the bioink along with biomaterials and growth factors, or they can be seeded later, after a supporting scaffold has been made with the bioink [59]. These cells can be of different types, such as fibroblasts, chondrocytes, stem cells, or epithelial cells, depending on the type of tissue to be made. During bioprinting, cells are deposited along with biomaterials that mimic the extracellular matrix, providing structural support. Once deposited, the cells must grow and differentiate in order to form functional tissue structures due to the presence of cytokines and growth factors [60,61].

The stem cells most commonly used for bone regeneration are bone mesenchymal stem cells (BMSCs), dental stem cells (DSCs), and adipose tissue-derived stem cells (ADCS) [62,63,64]. Following a bone defect, BMSCs migrate to the lesion and differentiate for the repair process. ADCSs are widely used cells due to several advantages, including their accessibility, the minimally invasive procedure for their collection, and the abundance of material. In addition, the latter have a remarkable capacity for osteogenic differentiation, and it has been shown that printing procedures do not affect their ability to differentiate and proliferate. Studies have shown how the combination of ADCS and polymers such as polyetheretherketone (PEKK) increases tissue integration and the process of osteogenesis [65,66,67,68].

## 5. Printing Techniques

There are several bioprinting techniques for making bioprinted constructs. The choice of technique depends on several factors, including the complexity of the bone construct to be made, the type of biomaterial used, and the mechanical properties required. The best bioprinting techniques for making bone tissue are several, and each of them has advantages and disadvantages (Table 2).

**Microextrusion bioprinting** is a technique that takes advantage of a hydride through which bioink is leaked. Mechanical forces are used to make the ink extrude, and three types of forces are employed: piston, pneumatic, and screw-driven. The advantages of this technique include versatility, i.e., the possibility of using different kinds of materials, even those with high viscosity, as well as cellular incorporation and scalability. Unfortunately, it is characterized by limited resolution, generally in the order of 100–500 µm, and by the mechanical stress that it can exert on cells, affecting their vitality [69,70].

**Inkjet bioprinting is a technique** that uses a head that deposits individual bioink droplets onto a substrate. The bioink droplets can be deposited by piezoelectric or thermal mechanisms. The materials that are used are low-viscosity, such as hydrogels and cell suspensions. Advantages of this technique include high printing speed, high resolution, even in the range of 20–100 µm, and minimal cellular stress. Unfortunately, this technique is limited to low-viscosity bioinks and is less suitable for large constructs [71].

**Laser-assisted bioprinting (LAB)** exploits a laser that generates pressure in order to propel bioink from a donor surface to a receiving substrate. To do this, the laser strikes the bioink-covered donor surface, generating a droplet that is deposited on the substrate. It is a technique that allows the fabrication of constructs of good mechanical strength, with relatively low cost, and is a simple process. The disadvantages of this technique are the high cost and the complexity of the printing process [72,73,74].

**Stereolithography bioprinting (SLA)** is a technique that uses a laser consisting of UV rays to polymerize a photosensitive fluid. It allows the fabrication of constructs with high resolution, in the range of 10–100 µm, speed, and accuracy. Unfortunately, this technique is limited to photosensitive resins, as UV rays can cause cell damage, affecting cell viability [75,76]. We have summarized the principal advantages and disadvantages associated with the most utilized bioprinting techniques, including microextrusion, inkjet, laser-assisted, and stereolithography methods, as shown in Table 2. The specific characteristics of each modality have dictated their suitability for creating the complex, heterogeneous structures required for maxillofacial bone regeneration.

## 6. Applications of Bioprinting for the Creation of Bone Constructs

Bioprinting is revolutionizing the way bone constructs are made. The applications of this technology are varied and include the potential repair of bone defects, often caused by traumatic, oncological, and degenerative diseases; the replacement of traditional autologous bone grafts, which while being the gold standard have limitations such as the limited availability of donor tissue and its morbidity; and the development of in vitro models for the study of bone disease and the testing of new pharmacological therapeutic strategies [77,78,79].

Regarding reconstructive surgery, particularly after resection of a portion of bone affected by neoplasm, such as the mandible, fibula flaps are used. Although the gold standard for jaw reconstruction, this surgical procedure is not without complications, particularly in terms of patient rehabilitation. The creation of bone tissue, while promising and fascinating, is not such a simple procedure considering that it is one of the most complex tissues to make [80]. Several studies have made bone bioprinted constructs by adopting different printing techniques and bioink compositions. We report some of the most recent ones in the literature (Table 3). The study conducted by Kérourédan et al. (2024) [81] made a biopaper using the laser-assisted printing technique capable of supporting vascularization in the bone construct.

The study conducted by Newby et al. (2023) [82] adopted a PLGA and graphene-based bioink combined with human mesenchymal stromal cells derived from adipose tissue with the aim of reconstructing a defect in rat femoral bone. This study highlights some limiting aspects of PLGA, including its hydrophobic property, poor mechanical integrity, and absence of osteogenic activity. In fact, PLGA can be combined with inducers of osteogenesis, such as hydroxyapatite or BMP2, conferring greater mechanical strength and bone regeneration capacity. In this case, graphene nanoparticles have proven useful in determining osteogenic response.

Another recent study by Li et al. (2024) [83] investigated an innovative extrusion printing technique, namely UV-assisted direct ink bioprinting, using a scaffold composed of hydroxyapatite and polyethylene-glycol-diacrylate (PEGDA) nanoparticles. This study demonstrated how this printing technique can meet certain properties required in the fabrication of bone tissue constructs, such as resistance to mechanical stresses and bone regeneration capability, while exhibiting good form integrity. In addition, this scaffold serves as a useful guide for the printing of biomaterials.

The study conducted by Seok et al. (2023) [84] also used a bioink consisting of a composition of different biomaterials, sodium alginate, and PCL, along with hPDC. In fact, it has been reported that the use of hydrogels alone as a base material has limitations and fails to meet the bifunctional characteristics that bone tissue requires. It was observed that the incorporation of PCL microparticles promoted a gradual and sustained release of BMP2, an important growth factor for cell differentiation. In addition, these microparticles resulted in increased cell adhesion, another key property for bioprinted constructs. Although the mechanical properties improved, the mechanical stress on the cells during printing affected cell viability, albeit to a lesser extent.

The study conducted by Zhang et al. (2024) [85] investigated a new methodology for the fabrication of vascularized bone tissue using triaxial bioprinting based on a mixture of GelMA bioink, sodium alginate, and various concentrations of MC3T3-E1 preosteoblasts encapsulated in β-TCP nanoparticles. A three-layer construct was made, the outermost layer consisting of GelMa, Alg, and proteoblasts with β-TCP, an intermediate layer consisting of GelMa, Alg, and human umbilical vein endothelial cells, and an inner layer consisting of gelatin as an expendable material for making canaliculi in order to simulate the microenvironment for bone and vascular tissues. They demonstrated how this technique provides good mechanical properties and excellent cell viability. The presence of β-TCP also determined increased osteogenic and angiogenic potential.

In addition to vascularization, a crucial aspect of tissue fabrication, the link between bone tissue and bone innervation has been highlighted and exploited. The study conducted by Wang et al. (2023) [86] made bioprinted constructs to facilitate bone regeneration by exploiting nerve-bone cross-talk. Indeed, peripheral nervous system stem cells contribute to the regulation of the microenvironment for bone regeneration and repair through paracrine actions. The key role is played by Schwann cell exosomes (SC-exosomes), which are characterized by their high content of let-7c-5p. They demonstrated how this molecule, present in exosomes, could regulate TGFβ signaling, a molecular signal crucial for regulating osteoblast differentiation, increasing bone regeneration by exploiting this naturally occurring mechanism. To illustrate the diversity and cutting-edge nature of these advancements, we have compiled a set of recent examples detailing novel material combinations, cell sources, and specific objectives achieved in the fabrication of bone constructs, as presented in Table 3. This synthesis highlights how researchers have leveraged advanced techniques and sophisticated cell-material interactions to move beyond simple structural scaffolds toward functional tissue engineering solutions.

## 7. Limits and Future Challenges

Bioprinting, although a cutting-edge and highly promising technology, still faces several challenges that limit its large-scale and clinical application. Among these, vascularization of bioprinted constructs remains the most critical. Functional vascular networks are essential for the survival and integration of engineered tissues, as they ensure oxygen and nutrient supply and remove metabolic waste. However, the current resolution of bioprinters is insufficient to reproduce capillary-sized vessels (~3 μm), with even advanced laser-assisted systems achieving around 20 μm. Other limiting factors include the biological and structural complexity of bone tissue, which requires the recreation of both its biomechanical and biological properties, as well as the high production costs and unresolved ethical and regulatory issues.

The bioprinting method exerts considerable mechanical and temperature stress on encapsulated cells, recognized as a critical factor influencing graft success. Extrusion bioprinting exposes cells to elevated shear stress as the bioink is propelled down the nozzle, which may result in acute cell membrane damage and apoptosis. We have observed that print resolution and speed must be meticulously managed to mitigate this hydrodynamic pressure. Moreover, the photo crosslinking phase has been identified in light-curing methodologies (e.g., SLA, DLP) as capable of producing lethal concentrations of reactive oxygen species (ROS), adversely affecting long-term functionality and differentiation potential.

Various ways have been devised and examined to enhance cell survival. The initial technique encompasses bioink formulation, wherein highly shear-thinning and low-viscosity substances have been engineered to facilitate extrusion at reduced pressures. The integration of protective chemicals, including antioxidants and growth hormones, directly into the bioink has demonstrated the ability to scavenge reactive oxygen species and enhance cellular recovery following printing. Moreover, the optimization of printing parameters has been shown to be crucial: decreasing the flow rate, enlarging the nozzle diameter, and lowering the printing temperature have all resulted in enhanced cell viability results. A tiered maturation process has been implemented after printing, using customized bioreactor conditions (e.g., dynamic flow, mechanical stimulation) to promote cell proliferation, differentiation into osteoblasts, and subsequent matrix remodeling.

### 7.1. Vascularization

The presence of a functional and stable vasculature is fundamental to any tissue, as it provides nourishment, defense, and metabolic exchange. Despite significant progress, reliable methods for achieving effective vascularization are still under development and represent one of the main focuses of current research [87].

Different cell types are used depending on the target tissue: CSCs for cardiac constructs, MSCs for bone and cartilage, iPSCs for cardiac, hepatic, neural, and osseous applications, and hCMPCs for vascularized cardiac tissue. Human umbilical vein endothelial cells (HUVECs) are among the most commonly employed for establishing vascular networks. In addition, several growth factors such as VEGF, βFGF, and HGF are known to promote neovascularization by stimulating endothelial cell activation and proliferation [88,89,90].

According to Mir et al. (2023) [91], the main obstacles in creating vascularized constructs include the instability of growth factors, the complexity of co-culture systems, and the difficulty of using synthetic materials to fabricate small, porous vessels.

Current bioprinting technologies still lack the resolution required to reproduce small vascular structures. The fabrication of functional capillaries would demand a precision of approximately 3 μm—a target not yet attainable, as even advanced laser-assisted bioprinters currently achieve resolutions around 20 μm. Another key aspect of vascularization concerns the crucial role of the endothelium, which serves as a selective barrier regulating metabolic exchange, inflammatory processes, and coagulation [92,93].

Despite significant progress in the vascularization of bone constructs, integrating vascular networks with osteogenic components remains a major challenge. Scaffold-based approaches and advanced hydrogel systems, such as modulated GelMA formulations, have demonstrated promising potential in forming interconnected vascular networks and promoting osteogenic differentiation. Moreover, emerging technologies such as volumetric bioprinting (VBP) offer new opportunities for the rapid fabrication of large, vascularized constructs. However, further advances in printing resolution and functional tissue integration are still required before clinical translation can be achieved [94].

### 7.2. Costs

3D bioprinting remains an expensive and technically demanding technology, requiring highly specialized expertise and sophisticated equipment. These factors currently limit large-scale production and clinical translation, particularly because each construct must often be customized for the individual patient. To enable broader adoption, future efforts should focus on streamlining manufacturing workflows, automating design and printing procedures, and developing standardized protocols that reduce preparation time and material waste. Advances in bioink formulation and printer hardware could also help lower production costs, while the implementation of open-source software for 3D modeling and CAD may enhance accessibility for smaller research centers. Ultimately, the combination of cost-efficient materials, modular printing systems, and shared bioprinting facilities could make this technology more scalable and economically sustainable in routine clinical practice [95,96].

### 7.3. Lead Times

Bioprinting could enable the fabrication of whole tissues and portions of organs in the future, which could be implanted for the surgical reconstruction of complex anatomical areas that have been demolished to remove tumor lesions. However, considering the lead time required to make the bioprinted constructs, the demand for tissue would not be met, and this turns out to be a major limiting aspect. In fact, the lengthy design and fabrication process limits the application in urgent clinical situations [97].

### 7.4. Biological–Structural Complexity, Printing Techniques, and Types of Materials

Bone tissue is one of the most difficult tissues to construct, compared to skin or cartilage tissue, precisely because of its structural and biological complexity. Not only is the correct shape of the construct required, but mechanical properties, such as resistance to stress, and biological properties, such as allowing the biological processes of bone regeneration and growth, must also be met. In addition, several printing techniques can be employed, each with its own advantages and disadvantages, such as UV printing or inkjet printing, which could affect the viability of the deposited cells. Another aspect to consider is the choice of materials to be adopted. In fact, several studies have already shown that using a single biomaterial is counterproductive for fabricating complex tissues like bone. To satisfy the multiple properties typical of native bone, blends of bioinks are often made [98,99].

In addition to traditional methods, two innovative modalities, 4D and volumetric bioprinting, have emerged as extremely promising approaches for the production of complex tissues. Additionally, 4D bioprinting, characterized as 3D printing integrated with a material that alters its shape over time (the fourth dimension) in reaction to external stimuli (e.g., pH, temperature, or hydration), has shown promise in producing patient-specific constructs capable of dynamically conforming to the intricate, irregular geometry of maxillofacial defects post-implantation. This intrinsic plasticity has provided benefits in attaining optimal fit and mechanical integration.

Volumetric bioprinting has evolved as a rapid, high-resolution technique. This method use light-sheet lithography to simultaneously cure an intricate 3D structure within a photo-sensitive bioink solution. The primary benefit has been the capacity to produce huge, intricately complex scaffolds in seconds, significantly minimizing cell exposure to cytotoxic agents and high shear stress, hence enhancing cell survival and scalability potential. This method has been specifically studied for establishing the complicated osteoid niche necessary for advanced bone tissue engineering.

### 7.5. Ethical and Regulatory Aspects

Bioprinting also raises ethical and regulatory issues, particularly regarding the use of stem cells and the fabrication of artificial human tissue. In addition, the customization of bioprinted grafts requires careful handling of sensitive data, considering privacy and data security concerns. Currently, regulations cannot handle the complexity of such technology, but it will be necessary to develop guidelines for process standardization, safety assessment, and product approval for clinical use [100].

Although bioinks must meet the criteria of printability and biocompatibility, significant obstacles persist about their preparedness for clinical application. A major concern has been on the possibility of a detrimental immunological response. Even extensively purified natural polymers (e.g., collagen, hyaluronic acid) are known to provoke mild inflammatory responses. The creation of genuinely immuno-inert or immunomodulatory bioinks has thus emerged as a significant research priority, with the objective of actively directing the host response towards regenerative healing instead of fibrosis.

The kinetics of long-term deterioration have posed a significant difficulty. The breakdown rate of the scaffold must exactly correspond to the rate of new bone growth. If the material deteriorates too rapidly, mechanical integrity is compromised before the defect is bridged; conversely, if it deteriorates too slowly, the scaffold remains and may obstruct the complete rebuilding of the native bone. We have emphasized the necessity for bioinks possessing adjustable, reliable degradation profiles to provide structural support throughout the prolonged healing period. The regulatory pathway has demonstrated complexity and difficulty. Bioprinted constructions, comprising cells, biomaterials, and a device, are subject to rigorous regulatory oversight (e.g., FDA or EMA). Manufacturers have encountered difficulties in proving lot-to-lot consistency, ensuring sterility, and establishing long-term stability of the live-cell component. The standardization of testing methodologies is acknowledged as a crucial measure for expediting clinical translation.

## 8. Diagnostic Implications

Accurate preoperative imaging and diagnostic evaluation are essential for the success of bone bioprinting and its translational application in maxillofacial surgery. High-resolution computed tomography (CT) and cone-beam CT (CBCT) enable precise quantification of bone defects, assessment of cortical and trabecular patterns, and volumetric modeling of the reconstruction site. These datasets serve as the foundation for Computer-Aided Design (CAD) and Computer-Aided Manufacturing (CAM) workflows, allowing the digital translation of anatomical information into printable scaffolds with patient-specific geometry [76].

Magnetic Resonance Imaging (MRI), particularly ultrashort echo time (UTE) and zero echo time (ZTE) sequences, has emerged as a complementary tool for evaluating bone marrow and vascular microarchitecture, supporting the selection of biomimetic materials that reproduce native mechanical anisotropy and vascular porosity [77].

After implantation of bioprinted bone constructs, imaging-based diagnostic follow-up is fundamental to monitor osteointegration, vascularization, and bone remodeling. Dynamic contrast-enhanced CT (DCE-CT) and perfusion MRI can visualize early neovascularization and graft perfusion. At the same time, micro-CT remains the gold standard for in vivo preclinical quantification of mineral density and trabecular microstructure [78].

Recent animal studies combining photoacoustic imaging with micro-CT demonstrated the feasibility of real-time monitoring of oxygenation and vascular maturation within GelMA/β-TCP bioprinted scaffolds, enabling early detection of hypoxic areas predictive of graft failure [79].

At the molecular level, diagnostic markers such as alkaline phosphatase (ALP), osteocalcin, and RUNX2 are used to evaluate osteoblastic differentiation within bioprinted tissues, whereas CD31 and VEGF immunostaining quantify neovascularization [80,81].

Recent transcriptomic profiling using spatial RNA sequencing on bioprinted bone grafts has identified early osteogenic signatures that correlate with CT-based mineralization indexes, supporting a multimodal diagnostic approach integrating imaging, molecular, and histological data [86].

Artificial intelligence (AI) is emerging as a powerful diagnostic and predictive tool in maxillofacial surgery. AI-based systems improve accuracy and reproducibility in image interpretation, segmentation, and surgical planning, supporting personalized and data-driven decision-making [87].

In bone regeneration research, several studies demonstrated a deep learning model capable of automatically segmenting bone grafts after maxillary sinus augmentation on CBCT scans, achieving high precision compared with manual analysis. Similarly, another study developed a neural network predicting implant osseointegration from plain radiographs, highlighting AI’s ability to detect early bone formation patterns [88,101].

These advances suggest that AI could play a key role in the diagnostic validation of bioprinted bone constructs, enabling automated quantification of graft integration and predicting osteogenic performance through imaging data analysis.

Recent studies have shown how machine learning can predict bioink behavior, control printing parameters in real time, and generate patient-specific bone scaffolds with ideal mechanical and vascular properties. These advances enhance reproducibility, reduce costs, and bring bioprinting closer to clinical translation [102,103,104].

Integration of multimodal diagnostic tools, combining CT-based morphometry, molecular imaging, and AI-driven predictive analytics, represents a cornerstone for translating 3D bioprinting into clinical practice. The establishment of diagnostic validation pipelines will enable quantitative assessment of graft performance, accelerate regulatory approval, and improve clinical outcomes in maxillofacial reconstruction.

The convergence of diagnostics, biofabrication, and computational modeling embodies the next step toward truly personalized bone bioprinting, aligning with the translational mission of modern maxillofacial surgery.

## 9. Discussion

3D bioprinting represents the new innovative frontier for regenerative medicine and tissue engineering. Such technology could revolutionize not only drug research with the study of new therapeutic strategies but also surgery. The hypothetical possibility of artificially creating tissues from an individual patient’s autologous cells would enable the implantation of portions of organs that are often surgically removed, particularly in patients with malignancy. This tool has the potential to be useful in facilitating tissue regeneration and reconstruction of complex anatomical areas.

Regarding maxillofacial surgery, there are several pathologies for which this technology would be beneficial. For example, oncological pathologies or bone defects due to trauma, infection, degenerative disease, or malformations. Nowadays, the gold standard for reconstructing anatomical areas is autologous flaps taken from the patient; bioprinting in the future could revolutionize this concept. There are already several studies analyzing, in vitro and in vivo, the fabrication of tissues such as skin, cartilage, mucosa, pulp-dentin complex, and bone on animals. The latter, most of all, proves to be the most complex due to its structural and biological-functional complexity. In fact, limitations persist even today that must be overcome in order to obtain a successful bioprinted bone construct that can be implanted in humans and thus be able to achieve clinical application of this technology [7,8].

The main obstacle to the fabrication of artificial bone tissue is vascularization. The creation of vascularized networks in bone tissue is a complex process. However, recent studies have applied and analyzed innovative methodologies for fabricating vascularized bone tissue using combined bioinks and new printing techniques. In addition, other limitations must be considered, such as cost, which prevents the large-scale diffusion of this technology, and the time required to make bioprinted constructs. In fact, application for emergency surgeries would be impossible at present, and also for surgeries where tumor lesions need to be removed, where time is crucial for the patient’s life. Additionally, it is essential to consider the ethical and regulatory aspects required to standardize processes, evaluate safety, and products for clinical use [105].

The most recent studies in the literature have applied different methods of making artificial bone tissue. To achieve the different physicochemical, biological, and mechanical properties of native bone tissue, it has been shown to be very fruitful to use combinations of multiple biomaterials loaded in bioinks, such as alginate combined with PCL or GelMA combined with β-TCP. In addition, natural biological processes, such as nerve-bone cross-talk, have been artificially applied to enhance bone regeneration. Creating a biolayer for in vitro pre-vascularization is also a promising technique for generating vascularized tissues [105].

Despite the strides made in research, multiple future challenges remain. One of the main challenges is the improvement of vascularization. The development of new combined bioinks and the use of growth factors would also seem to be the correct way forward to print tissues that can meet the properties of native tissues. Another area of focus for research is the development of innovative techniques, such as 4D bioprinting. This technology would allow bioprinted constructs to change their morphology over time, facilitating and improving the post-bioprinting phase in which tissue maturation occurs. Another innovative technology is volumetric bioprinting, which enables rapid printing of complex structures, meeting the need for faster printing times. Another challenge is the integration of bioprinting with imaging techniques and 3D modeling. Indeed, the advanced use of imaging data, such as CT or MRI scans, and 3D design would allow the fabrication of highly personalized bioprinted constructs specific to the individual patient [106,107].

The clinical translation of bioprinting also raises complex regulatory and ethical challenges. Standardizing manufacturing processes, ensuring reproducibility, and establishing quality-control guidelines are essential steps before patient-specific bioprinted tissues can be approved for clinical use. Ethical considerations include the origin and manipulation of human cells, informed consent for cell sourcing, and long-term monitoring of implanted constructs. From a regulatory standpoint, collaboration between academic institutions, industry, and governing agencies is crucial to define safety, validation, and certification pathways. At the same time, progress toward functional vascular networks will depend on optimizing current strategies—such as combining angiogenic growth factors with perfusable scaffolds and microfluidic designs—while reducing costs through automation, reusable bioinks, and shared bioprinting platforms. The convergence of these innovations will mark the trajectory toward clinically viable, scalable, and ethically responsible bioprinting applications in regenerative maxillofacial surgery [100,105]. In addition, Hajaj et al. has shown that Zirconia fixed partial dentures provided important data on the biomechanical requirements and longevity of permanent, non-cellular dental restorations, which is directly relevant to the long-term clinical outcome and functional demands of the maxillofacial region [108].

## 10. Conclusions

In conclusion, 3D bioprinting is a cutting-edge field in tissue engineering and regenerative medicine that may find use in surgery and pharmaceutical research. The development of artificial tissues might enhance the rebuilding of intricate anatomical regions and lessen the necessity for major surgical excisions. This technology may provide innovative treatments for bone abnormalities, infections, malformations, injuries, and cancer in maxillofacial surgery. Major challenges still exist despite recent advancements, including the development of functional vascular networks in bone tissue, the time and expenses involved in designing and producing the bioprinted product, and the ethical and legal concerns that must be resolved in future studies before this technology can be used in clinical settings. The development of novel composite biomaterials, pre-vascularization, 4D and volumetric bioprinting, and integration with imaging and 3D modeling techniques are some of the topics that require further research to enhance the quality of bioprinted tissues.

## Figures and Tables

**Table 1:** Advantages and disadvantages of the principal types of treatment for bone defects.

Types of Treatment	Advantages	Disadvantages
Autologous bone graft	Gold standard; Excellent biocompatibility; Low rejection risk; Rapid ossification and bone regeneration.	Donor site morbidity; Limited bone availability; Technically demanding.
Allogenic bone transplantation	Readily available; No donor site; Suitable for poor bone reserve.	Risk of rejection/infection; Possible resorption; Disease transmission risk.
Metal prostheses (e.g., titanium)	High mechanical strength; Immediate stability; Long-lasting; Customizable (3D printing).	Low biocompatibility; Long-term complications; Limited compatibility with some techniques.

**Table 2:** Advantages and disadvantages of the main bioprinting techniques.

Printing Techniques	Advantages	Disadvantages
Microextrusion bioprinter [69,70]	Wide range of materials; Cellular incorporation; Even large constructs.	Limited resolution; Mechanical stress.
Inkjet bioprinter [71]	High speed; High resolution; Minimal cellular stress.	Limited materials; Only small constructs.
Laser-assisted bioprinter [72,73,74]	High precision; Minimal cellular stress; Wide range of materials.	High cost; Complexity.
Stereolithography bioprinter [75,76]	High resolution; Good speed; Accuracy.	Limited materials; UV Toxicity.

**Table 3:** Examples of new methodologies for creating bioprinted bone constructs.

	Materials	Cells/Molecules	3D Printer	Objective/Result
Kérourédan et al. (2024) [81]	Gelatin + bioactive glasses (BGs)	Human umbilical vein endothelial cells (HUVEC)	Laser-assisted bioprinter (LAB)	Fabrication of vascularized bone constructs.
Newby et al. (2023) [82]	PLGA + graphene	Mesenchymal stromal cells derived from adipose tissue	Microextrusion bioprinter	Rat femoral bone defect reconstruction.
Li et al. (2024) [83]	Hydroxyapatite + polyethylene-glycol-diacrylate (PEGDA)	N/A	UV-assisted extrusion bioprinter	Evaluation of optimal parameters. Demonstrated how the printing process is accurate, repeatable, and with excellent controllability. Additionally, it possesses good mechanical strength and bone regeneration ability.
Seok et al. (2023) [84]	Sodium alginate + PCL microparticles	hPDC/BMP2	Extrusion bioprinter	PCL microparticles increased cell adhesion and ensured a continuous supply of growth factor, resulting in sustained release of BMP2.
Zhang et al. (2024) [85]	Gelatin + GelMA + Alginate	MC3T3-E1 preosteoblasts encapsulated in nano β-TCP + human umbilical vein endothelial cells (HUVEC).	Fused deposition bioprinter.	Fabrication of vascularized bone tissue using a triaxial bioprinting system.
Wang et al. (2023) [86]	GelMa + SiMA	BMSCs + Schwann cell exosomes	N/A	Fabrication of bioprinted constructs to facilitate bone regeneration by exploiting nerve-bone cross-talk.

## Data Availability

No new data were created or analyzed in this study. Data sharing is not applicable to this article.
